# Genome-wide identification and characterization of DCL, AGO and RDR gene families in sister mangrove species *Kandelia obovata* and *Kandelia candel*

**DOI:** 10.1016/j.bbrep.2025.102197

**Published:** 2025-08-02

**Authors:** Runqi Zhang, Bonian Shui, Zihui Jin, Chenxi Cui, Shiqi Zhao

**Affiliations:** a School of Fishery, Zhejiang Ocean University, Zhoushan, 316022, China; b Zhejiang Marine Fisheries Research Institute, Zhoushan, 316021, China

**Keywords:** Transcriptional silencing, Argonaute, Dicer-like, RNA-Dependent RNA polymerase, Mangrove

## Abstract

The AGO, DCL and RDR gene families work together in the plant gene silencing process, playing crucial roles in regulating gene expression, responding to stress, and gene silencing, thus forming an essential part of the plant's defense system. In this study, we systematically identified and characterized these genes in the closely related mangrove species *Kandelia obovata* and *Kandelia candel*. We found a total of 8/6 AGO, 4/3 DCL and 5/4 RDR genes in these species, respectively. Phylogenetic analysis classified them into 3, 4, and 4 subfamilies correspondingly. Comparing with the sister species *K. candel* helped clarify key genes involved in cold tolerance. Each gene exhibited different expression levels across various tissues and stress conditions, with certain genes showing notably high expression. Furthermore, analyses of promoters, protein interaction networks, and three-dimensional structures provided deeper insights into the possible reasons behind the greater cold tolerance of *K. obovata* compared to *K. candel*. These comprehensive results offer a theoretical basis for further experimental validation of the specific roles of these genes in *K. obovata*'s stress resistance and for investigating gene-environment interactions.

## Highlights

• First *K. obovata*-*K. candel* AGO/DCL/RDR genome comparison.
• Compare to *K. candel*, *K. obovata* subfamily expansions.
• Some gene are crucial for *K. obovata* stress response.

## 1 Introduction

The AGO(Argonaute), DCL(Dicer-like) and RDR (RNA-dependent RNA polymerase) gene families are key components of the RNA interference (RNAi) pathway, which regulates gene expression in plants, animals and fungi [1,2]. These families play essential roles in antiviral defence, developmental processes and stress responses. They play pivotal roles in small RNA (sRNA) biogenesis and gene silencing. Central to the RNA-induced silencing complex (RISC), AGO proteins bind small RNAs such as microRNAs (miRNAs) and small interfering RNAs (siRNAs) to target messenger RNAs (mRNAs), leading to mRNA degradation or translation inhibition [3]. AGO proteins possess PAZ and PIWI domains; the latter exhibit endonuclease activity that directly mediates gene silencing and aids viral resistance [4]. DCL proteins cleave long double-stranded RNA (dsRNA) into small RNAs, including miRNAs and siRNAs. Different DCL members are involved in various RNA processes: DCL1 is involved in the biogenesis of miRNAs, DCL3 is involved in the production of developmental siRNAs, and DCL4 is involved in antiviral responses [5]. RDR proteins amplify RNAi signals by synthesising dsRNA from ssRNA. This ‘RNA amplification effect' is crucial in processes such as antiviral immunity and chromatin silencing, with RDR6 playing a key role in enhancing antiviral siRNA accumulation [6]. Together, these gene families regulate gene expression and stress resistance through the RNAi pathway, thereby influencing plant development and environmental adaptability [7].

*Kandelia obovata*, a species of mangrove in the Rhizophoraceae family, plays a crucial role in coastal ecosystems [8], offering ecological benefits like shoreline stabilization and water purification. Despite their tropical origin, mangroves such as *K. obovata* face challenges from low-temperature stress at their northern distribution limits [9]. As a sister species to *K. obovata*, *K. candel*, which is distributed further south, offers a natural advantage in the study of *K. obovata* evolution [10]. Recent conservation efforts, including China's national protection and restoration programmes, have led to an overall increase in mangrove areas, emphasising their significance as ecological treasures and carbon sinks [11]. *K. obovata* exhibits cold tolerance, especially through specific microRNAs that regulate key genes involved in cold resistance [12,13]. Understanding its molecular response mechanisms is essential for improving mangrove conservation and facilitating the northward migration of this species. Low temperatures significantly affect *K. obovata*, inhibiting its growth and physiological processes [14]. Temperature stress leads to reduced photosynthesis, membrane damage and decreased chlorophyll content, impairing nutrient transport and overall growth [[15], [16], [17], [18]]. However, *K. obovata* initiates protective responses such as osmotic adjustments and antioxidant enzyme activity to mitigate damage [16]. Molecularly, cold-induced genes like CBF play key roles in regulating cold tolerance [12]. Therefore, studying the physiological and molecular mechanisms of the cold stress response is crucial for enhancing K. obovata's cold adaptation and informing its conservation and genetic improvement.

This study aims to identify and analyze the AGO, DCL and RDR gene families in *K. obovata* and *K. candel*, focusing on their roles in responding to low-temperature stress, Waterlogging and Saline-Alkali Stress. Although these gene families have been studied in other plants, research on *K. obovata* remains limited especially evolutionary comparisons with plants of the same genus are rarely seen. By exploring the molecular characteristics and expression patterns of these genes, this research will enhance our understanding of *K. obovata*'s environmental adaptability and contribute to improving its cold resistance, with implications for mangrove conservation and climate change adaptation.

## 2 Materials and methods

### 2.1 Acquisition and identification of AGO, DCL and RDR genes

Firstly, the HMM files for the AGO, DCL and RDR gene families were downloaded from the Pfam database (http://pfam.xfam.org/) [19]. The correctness of the obtained AGO, DCL and RDR gene sequences was assessed by checking whether the structural diagrams generated by the SMART website contained the following domains: for AGO proteins, the ArgoMid domain (PF16487.4), ArgoN domain (PF16486.4), PAZ domain (PF02170.21), and Piwi domain (PF02171.16); for DCL proteins, the DEAD domain (PF00270.28), Dicer_dimer domain (PF03368.13), Helicase_C domain (PF00271.31), PAZ domain (PF02170.21), and Ribonuclease domain (PF00636.25); and for RDR proteins, the RdRP domain (PF05183.11). Subsequently, the genomic sequence and annotation files for *K. obovata* and *K. candel* were obtained from the NCBI database (https://www.ncbi.nlm.nih.gov) [20]. The HMMER search tool in TBtools was then used to identify these genes from their genome [21]. Finally, the predicted these genes were validated using the SMART website (https://smart.mbl.de/) and BLAST. For the validated genes, the corresponding gene IDs, complete genomic sequences, and the start and end positions of these genes on the chromosome were searched in the annotation file using their protein IDs. Additionally, amino acid sequence accession numbers for the these genes from *Arabidopsis thaliana*, *Vitis vinifera*, and *Glycine max* were searched in the literature or databases, and relevant data were downloaded from the NCBI database for comparative analysis late [22].

### *2.2* *Sequence analysis of AGO, DCL and RDR genes in K. obovata* and *K*. *candel*

First, the SMART website (https://smart.mbl.de/) was utilized to predict and analyze the conserved domains of the AGO, DCL and RDR protein sequences in them. Additionally, the MEME Suite website(https://meme-suite.org/meme/) was used to visualize the conserved protein domains of the these gene families in *K. obovata* and *K. candel* [23]. It is important to note that some domains may not be displayed on the SMART website due to formatting issues; therefore, it is necessary to check for omissions in the section titled “Features NOT shown in the diagram” at the bottom right of the page to ensure the accuracy of subsequent analyses. Next, the TBtools Visualize Gene Structure (Basic) tool was employed to create distribution maps of the exons and introns for each gene in *K. obovata*. Finally, the Expasy website (https://web.expasy.org/compute_pi/) was used to calculate the length and isoelectric point of these genes sequences.

### 2.3 Construction of phylogenetic trees for representative species

The protein sequences of AGO, DCL and RDR from representative species including *K. obovata*, *K. candel*, *A*. *thaliana*, *G*. *max*, and *V*. *vinifera* were combined into separate files representing the three gene families. These files were then imported into MEGA11 [24], selecting the alignment option for file opening. The aligned amino acid sequences were processed using the MUSCLE algorithm for protein alignment with default parameters, and the files were saved. Subsequently, in the Analysis section, the Neighbor-Joining method was selected to open the recently saved file. The parameters were set with the number of Bootstrap Replications at 1000, the model selected as the Dayhoff model, Rates among Sites set to Uniform Rates, and Gap/Missing Data Treatment chosen as Pairwise deletion. The evolutionary tree shape was specified, and MEGA software was used to categorize the phylogenetic tree. Finally, the evolutionary tree was beautified using Adobe Illustrator 2023.

### 2.4 Expression analysis of AGO, DCL and RDR in K. obovata under different stress and tissues

First, raw transcriptome data for different tissues of *K. obovata* (including leaf, stem, flower, pistil, root, stamen, sepal, and fruit) and under various stress conditions (including low temperature, saline-alkali, and waterlogging) were downloaded from the NCBI SRA database. Following quality assessment using FastQC (v0.11.9) [25], the downloaded data were confirmed to consist of clean reads, with adapters and low-quality reads removed, suitable for further analysis. Subsequently, Hisat2 (v2.2.1) [26] was used to align the data to the *K. obovata* genome. FeatureCounts (v2.0.3) was then employed to quantify gene expression levels in TPM (Transcripts Per Million) for each gene, using default parameters. After extracting the expression data for the *K. obovata* genes, the data were imported into TBtools. The Heat Map tool within TBtools was utilized to generate heat maps, with expression values normalized.

### 2.5 The promoter analysis of AGO, DCL and RDR genes in K. obovata

First, the GXF Sequences Extract function in TBtools was used to initialize the GFF file and sequence file of *K. obovata*, selecting only the CDS upstream 2000 bp. The resulting files were then checked using Fasta Stats. The Fasta Extract tool was used to process the files and extract the sequence information of the required genes. Subsequently, promoter analysis was conducted using the PlantCARE website (https://bioinformatics.psb.ugent.be/webtools/plantcare/html/). The files obtained in the previous step were submitted to the Search section, and the results were downloaded. After opening and processing the tab file, only the gene IDs, locations, and annotations were retained, and the target elements were manually selected. Finally, the modified tab file was processed using Basic Biosequence View to generate the promoter visualization of the K. obovata AGO, DCL and RDR genes.

### 2.6 Protein-protein interaction analysis of K. obovata with model organisms

The protein interaction analysis was conducted using the STRING database by importing the sequence file and selecting *A*. *thaliana* for the search. After selecting proteins with relatively high confidence, the next step is to click on “Continue”. The obtained interaction network was then edited and saved for further analysis.

### 2.7 Prediction and Analysis of the tertiary structure of K. obovata

The amino acid sequence of *K*. *obovata* to be analyzed was input into the SWISS-MODEL website, and “Search For Templates” was selected. The tertiary structure model with the highest similarity was chosen for modeling. After adjusting and optimizing the structure, the model was saved for subsequent analysis.

## 3 Results

### *3.1* *Identification and structural analysis of the AGO, DCL and RDR in K. obovata* and *K. candel*

**Fig. 1:**
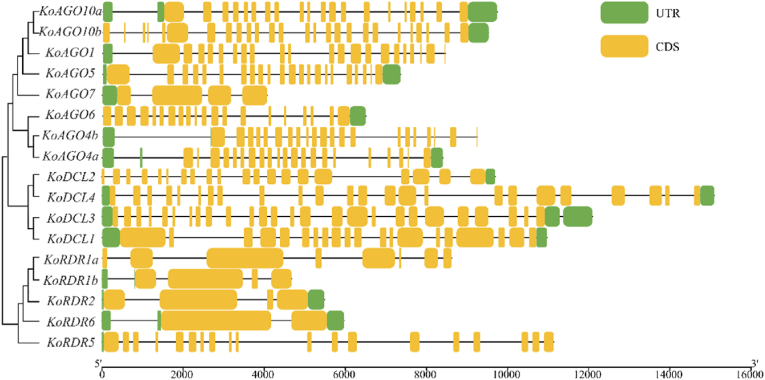
Exon-intron structure diagram of AGO, DCL and RDR genes in K. obovata.

The identification of the AGO, DCL and RDR gene families in *K. obovata* and *K. candel* revealed a total of 14 AGO genes (Table 1). Based on clustering analysis, these genes were classified into the AGO1, AGO2 and AGO4 groups. They are located in the nucleus, the chloroplast and the cytoplasm. Additionally, 7 DCL genes were identified, which correspond to DCL1, DCL2, DCL3, and DCL4, respectively. These genes are distributed in the nucleus, the plasma membrane and the chloroplast. Furthermore, 9 RDR genes were identified, belonging to RDR1, RDR2, RDR3, and RDR6, respectively. Their locations include the plasma membrane, cytoplasm, chloroplast and nucleus. By analyzing protein instability coefficients, we observe that the evolution of *K. obovata* and *K. candel* shows a considerable degree of convergence. Specific proteins, such as DCL1, RDR1a, and RDR6, exhibit high stability in both species, which suggests that these proteins play a central role in the RNAi (RNA interference) pathway. Moreover, the gene sequences of these stable proteins are highly similar between the two sister species, indicating that under prolonged environmental pressures, these genes have maintained critical functions both before and after species divergence, which likely explains why they have not been eliminated. The majority of other proteins exhibit moderate stability, enabling them to achieve dynamic regulation through flexible degradation mechanisms, thus allowing adaptation to varying environmental demands (Table 1). *K. obovata* ’s exon-intron distribution also varies (Fig. 1), AGO family members (excluding *KoAGO7*) exhibited similar exon numbers, while DCL genes were generally longer, with *KoDCL4* exceeding 15,000 bp in length. In contrast, RDR genes had the smallest number of exons and were also the shortest in terms of gene length among the three families.

**Table 1:** Basic physicochemical properties of AGO, DCL and RDR gene families in *K. obovata* and *K. candel.*

Name	Gene-ID	Class	Exon	PI	Protein	Subcellular	Instability
			No.		Length(aa)	Localization	Index
*KoAGO1*	GWHGACBH008992	AGO1	22	9.51	1094	nucl	52.33
*KoAGO4a*	GWHGACBH018135	AGO4	22	9.18	823	nucl	50.97
*KoAGO4b*	GWHGACBH012002	AGO4	22	8.90	850	nucl	47.32
*KoAGO5*	GWHGACBH015456	AGO1	23	9.58	976	chlo	39.85
*KoAGO6*	GWHGACBH014001	AGO4	21	9.24	902	nucl	40.32
*KoAGO7*	GWHGACBH006686	AGO2	5	9.38	915	nucl	48.97
*KoAGO10a*	GWHGACBH007846	AGO1	21	9.24	945	chlo	47.25
*KoAGO10b*	GWHGACBH012765	AGO1	27	9.21	1120	chlo	46.97
*KoDCL1*	GWHGACBH013660	DCL1	21	5.93	1973	nucl	39.73
*KoDCL2*	GWHGACBH008434	DCL2	21	7.70	1341	plas	43.73
*KoDCL3*	GWHGACBH015050	DCL3	27	6.84	1642	nucl	41.63
*KoDCL4*	GWHGACBH001467	DCL4	25	6.09	1553	nucl	48.26
*KoRDR1a*	GWHGACBH004476	RDR1	9	8.22	1364	plas	39.25
*KoRDR1b*	GWHGACBH004732	RDR1	5	6.31	1007	cyto	41.91
*KoRDR2*	GWHGACBH000368	RDR2	5	6.48	1115	cyto	40.46
*KoRDR5*	GWHGACBH014890	RDR3	20	6.17	1029	nucl	46.83
*KoRDR6*	GWHGACBH004103	RDR6	3	7.81	1197	nucl	34.57
*KcAGO1*	Kcandel.15559.1|m.4512	AGO1	21	9.51	1063	nucl	51.66
*KcAGO2*	Kcandel.15297.1|m.4428	AGO2	2	9.20	1026	cyto	34.32
*KcAGO4*	Kcandel.12388.1|m.3390	AGO4	23	914	912	nucl	49.38
*KcAGO5*	Kcandel.10694.1|m.4929	AGO1	21	9.52	1043	nucl	41.24
*KcAGO10a*	Kcandel.1059.1|m.4569	AGO1	21	9.18	991	chlo	47.54
*KcAGO10b*	Kcandel.4088.2|m.3245	AGO1	21	9.17	991	chlo	49.73
*KcDCL1*	Kcandel.6035.1|m.589	DCL1	19	5.91	1973	chlo	39.99
*KcDCL2*	Kcandel.15116.2|m.2102	DCL2	21	7.75	1441	plas	45.14
*KcDCL4*	Kcandel.3060.1|m.1214	DCL4	24	5.94	1633	nucl	49.48
*KcRDR1a*	Kcandel.11960.1|m.2103	RDR1	4	7.32	1124	chlo	37.44
*KcRDR1b*	Kcandel.155.1|m.3265	RDR1	4	6.22	1125	nucl	41.03
*KcRDR2*	Kcandel.2148.1|m.3995	RDR2	3	6.66	1142	chlo	40.01
*KcRDR6*	Kcandel.13659.1|m.2531	RDR6	2	7.94	1197	cyto	34.41

### 3.2 Phylogenetic analysis of AGO, DCL and RDR in K. obovata and K. candel

**Fig. 2:**
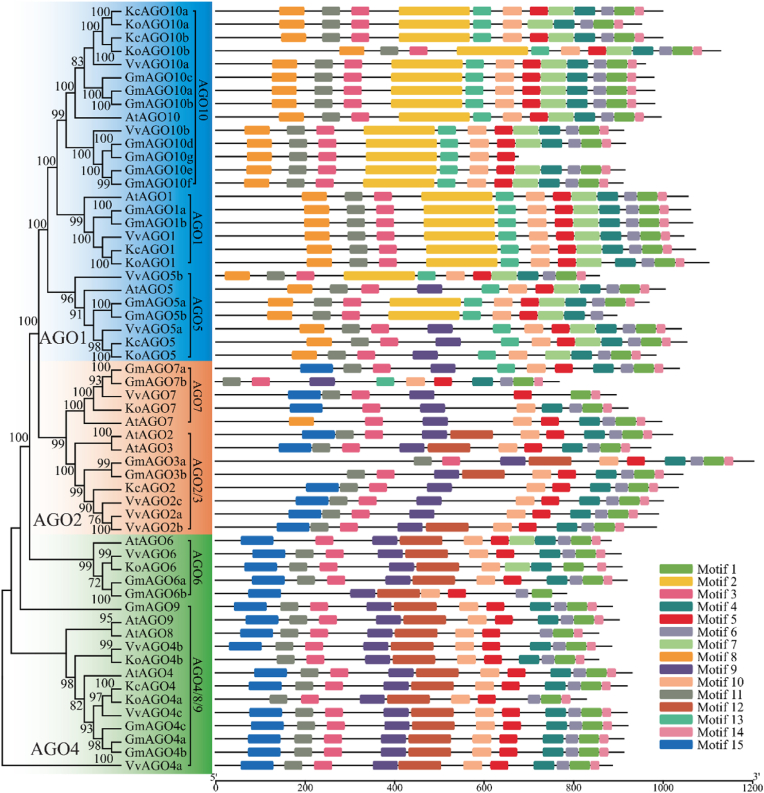
Phylogenetic Tree and Conserved Domains of AGO Gene Family in *K. obovata* and *K. candel*. Different color blocks represent different subfamilies, and different motifs are represented by different colors.

**Fig. 3:**
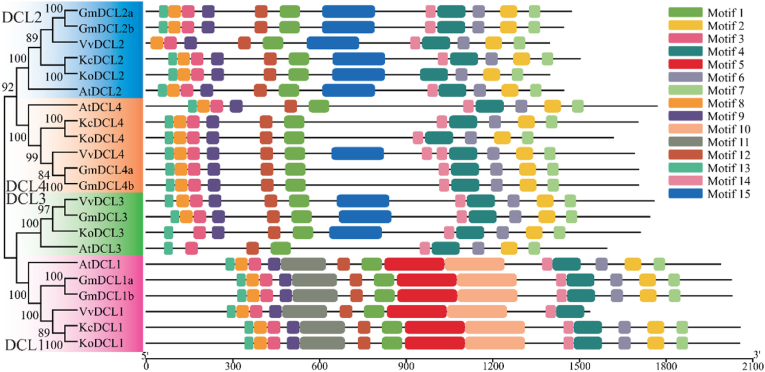
Phylogenetic Tree and Conserved Domains of DCL Gene Family in *K. obovata* and *K. candel*. Different color blocks represent different subfamilies, and different motifs are represented by different colors.

**Fig. 4:**
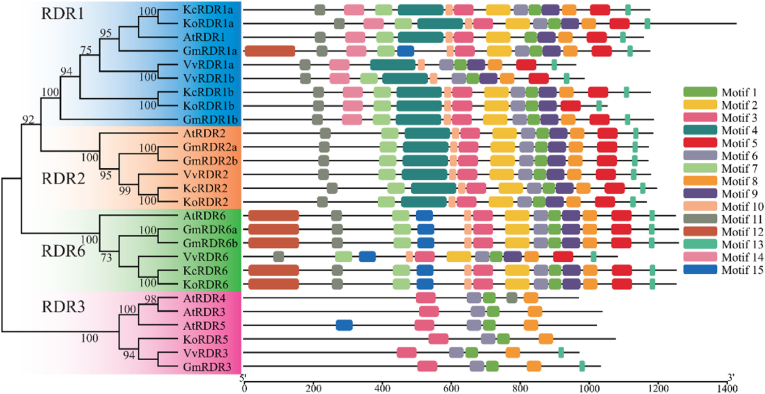
Phylogenetic Tree and Conserved Domains of RDR Gene Family in *K. obovata* and *K. candel*. Different color blocks represent different subfamilies, and different motifs are represented by different colors.

Structural predictions of the AGO, DCL and RDR protein sequences were performed using the SMART website. The evolutionary tree and protein conserved domain diagrams for these gene families in *K. obovata* and *K. candel* were generated using the MEME Suite and TBtools Graphics BioSequence Structure Illustrator Gene Structure View (Advanced) (Fig. 2, Fig. 3, Fig. 4). The results indicated that all AGO genes possess the ArgoMid, ArgoN, PAZ, and Piwi domains; all DCL genes contain the DEAD, Dicer_dimer, Helicase_C, PAZ, and Ribonuclease domains; and all RDR genes have the RdRP domain. A total of 58 AGO genes, 22 DCL genes, and 27 RDR genes were identified from *K. obovata*, *K*. *candel, A*. *thaliana*, *V*. *vinifera*, and *G*. *max*. The AGO genes can be classified into three subfamilies: AGO1, AGO2, and AGO4. These subfamilies can be further subdivided into AGO1, AGO5, and AGO10 (AGO1); AGO2/3 and AGO7 (AGO2); and AGO4/8/9 and AGO6 (AGO4). Except for AGO2/3, *K. obovata* has representatives in all other members, which may indicate gene contraction [27]. In the AGO gene family of *K. obovata* and *K. candel* (Fig. 2), *KoAGO1*, *KoAGO10b*, *KcAGO1*, *KcAGO5*, *KcAGO10a* and *KcAGO10b* has the most domains, totaling 12, *KoAGO7* has least, only 8. Phylogenetic analysis shows that they are mostly similar in domain structure to proteins from other plants within the same category, indicating that their regulatory functions or roles may be similar. However, some gene proteins, such as *KoAGO4b* and *KoAGO5*, are missing certain structures compared to others, which may relate to their specific functions. The longest protein is 1120 amino acids (*KoAGO10b*), while the shortest is only 823 amino acids (*KoAGO4a*). The isoelectric points of these genes are quite close, and except for *KoAGO7* and *KcAGO2* the number of exons is also similar. In the DCL gene family of them (Fig. 3), the diversity of the genes is more pronounced. *KoDCL1*, *KcDCL1* has the most domains at 14, other has 11 domains. The diversity in the DCL gene family suggests that these genes may participate in numerous regulatory process [28]. During *K. obovata*'s response to environmental stimuli, the DCL gene family likely plays a crucial and active role. The longest protein is 1973 amino acids (*KoDCL1*, *KcDCL1*), and the shortest is only 1341 amino acids (*KoDCL2*). Based on isoelectric points, they are mostly composed of acidic amino acids (except for *KoDCL2*). In the RDR gene family of them (Fig. 4), most genes have a total of 12–13 protein domains, while *KoRDR5* has only 4 domains, which may be related to their different functions. Additionally, although *KoRDR1b*, *KcRDR1b* and *KoRDR1a*, *KcRDR1a* are classified in the same category, their number of protein domains differs. This distinction, along with differences observed in other plants within the same group (*GmRDR1*, *VvRDR1*), may be associated with their differential expression or functional variations. The isoelectric points and the number of exons show significant variation, likely due to their localization in different cellular structures and their differing functions [29].

*K. obovata*, *K*. *candel, A*. *thaliana*, *V*. *vinifera*, and *G*. *max* possess nearly identical genes and are closely related, originating from a common ancestor. This allows for further inference about the evolutionary relationships and differences of the AGO gene family among these species, with a clustering coefficient of 100 indicating the closest phylogenetic relationships. Based on this, smaller numbers indicate more distant relationships. Among the three subfamilies, the AGO1 subfamily contains the most AGO genes, totaling 27, including the AGO1, AGO5, and AGO10 genes from *K. obovata*, *K*. *candel, A*. *thaliana*, *V*. *vinifera*, and *G*. *max*. The AGO4 subfamily is relatively independent, with *KoAGO4a* and *KoAGO4b* belonging to different branches, unlike *KoAGO10a* and *KoAGO10b*. This may be due to gene duplication events during the evolutionary process of *K. obovata*, leading to different branches because of varying selective pressures, although the possibility of parallel evolution cannot be ruled out [30]. The DCL genes are divided into four subfamilies: DCL1, DCL2, DCL3, and DCL4. These subfamilies are relatively independent, and almost all species are represented in these four subfamilies. Interestingly, not all genes within the same subfamily exhibit high similarity. The similar distribution of DCL genes among *K. obovata*, *K*. *candel, A*. *thaliana*, *V*. *vinifera*, and *G*. *max* suggests that the subfamilies of this gene family may have critical functions that have been conserved throughout evolution. The independence of subfamilies and their differentiation patterns not only highlight the functional diversity and ancient nature of the family but also indicate that independent evolution plays an important role in maintaining these subfamilies, allowing different DCL genes to function under various environmental or physiological conditions. The phylogenetic situation of RDR genes is similar to that of DCL genes, also divided into four subfamilies: RDR1, RDR2, RDR3, and RDR6, with relative independence among subfamilies and distribution across species. In contrast, within the RDR1 subfamily, *KoRDR1b* and *KcRDR1b* has formed a distinct branch, indicating that they may have undergone a unique evolutionary pathway, accumulating a significant number of mutations that lead to notable differences compared to other genes in the family. Alternatively, they may have acquired new functions after duplication, experiencing different selective pressures that gradually diverged it from the evolutionary trajectories of other genes.

*K. obovata* and *K. candel*, sister species within the *Kandelia* genus, share many evolutionary similarities; however, due to their different geographic distributions, they also exhibit certain differences [31]. Within the AGO2 and AGO4 subfamilies, there are some evolutionary divergences between the two species, while in the DCL and RDR gene families, *K. obovata* has evolved an additional *KoDCL3* and *KoRDR5* compared to *K. candel*. These evolutionary differences may be an important factor contributing to the greater cold tolerance of K. obovata compared to K. candel [32].

### 3.3 Analysis of the Types and Distribution of Promoter *Cis*-acting elements in K. obovata

**Fig. 5:**
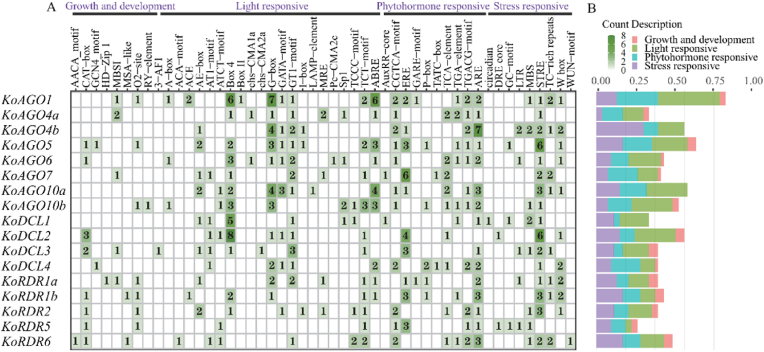
Analysis of the Types and Distribution of Promoter *Ci*s-acting Elements in *K. obovata*. Green squares indicate the number of *cis*-acting elements per gene (0–8, light to dark gradient). Color codes: pink (Growth and development), cyan (Light responsive), indigo (Phytohormone responsive), purple (Stress responsive). The x-axis represents the proportion of these four categories in each gene.

We identified a total of 396 *cis*-acting elements across the AGO, DCL and RDR gene families in *K*. *obovata*. We specified that each member was classified into four groups: Growth and development, Light responsive, Phytohormone responsiveness, and stress responsive (Fig. 5). Among these four groups, Light responsive elements were the most prevalent, while Growth and development had the fewest. Almost all members contain these four elements, with the exception of *KoAGO4b*, *KoAGO10a*, and *KoDCL1*. In addition, we found that elements related to plant growth and development accounted for the least proportion of AGO, DCL and RDR gene family members in *K. obovata*. Some genes, such as *KoAGO4b*, *KoAGO5* and *KoDCL2*, have a relatively high proportion of stress-related elements, which suggests that they may play an important role in the response to low temperature stress.

Among these genes, *KoAGO1* contained the highest number of *cis*-acting elements (44), while *KoRDR5* had the fewest (14). Additionally, *cis*-acting elements involved in defense and stress responses, abscisic acid responsiveness, and anaerobic induction were identified. Under varying environmental conditions, these promoters interact through multiple signaling pathways to form complex regulatory networks, playing critical roles in plant growth and development, stress resistance, photosynthesis, and pest resistance [33]. Cold-responsive *cis*-acting elements were detected in *KoAGO4b*, *KoAGO6*, *KoDCL3*, *KoRDR1a*, and *KoRDR5*. Under low-temperature conditions, these genes express cold-tolerance-related genes, enhancing the cold hardiness of *K. obovata*. Furthermore, auxin-responsive elements and *cis*-acting elements associated with abscisic acid responsiveness may regulate gene expression by modulating hormone levels through their interactions, thereby further improving the cold tolerance of *K. obovata*.

### 3.4 Expression of AGO, DCL and RDR genes in different tissues and stress in K. obovata

**Fig. 6:**
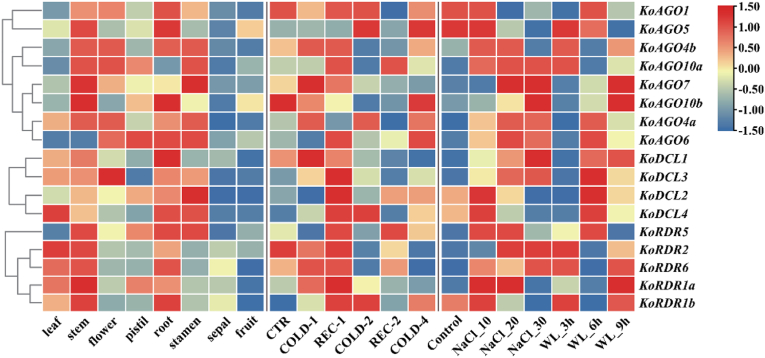
Expression of AGO, DCL and RDR Gene Families in Different Tissues and Stress of *K. obovata.* “a” is the expression of these three gene families in different tissues of *K. obovata*; “b” is the expression of these three gene families under low temperature stress. “c” is the expression of these three gene families under saline-alkali stress; “d” is the expression of these three gene families under the waterlogging stress.

We analyzed the differential expression of *K. obovata* genes across various tissues and under different stress conditions (low temperature, saline-alkali, and waterlogging) (Fig. 6). In the AGO gene family, differential expression among the members across tissues was highly significant. *KoAGO5* and *KoAGO10b* exhibited high expression in both roots and stems, suggesting their potential roles in root development and stress adaptation. In contrast to other members, *KoAGO4a* also showed some expression in leaves. *KoAGO1*, *KoAGO4a*, *KoAGO4b*, *KoAGO6*, and *KoAGO10* exhibited broad expression patterns, highlighting their potential functional significance [34]. Unlike other members of the DCL gene family, *KoDCL3* displayed high expression across many tissues but showed specific and significant expression in flowers. Interestingly, almost all members of the RDR gene family exhibited high expression in leaves, a unique feature of the RDR family compared to the other two families, suggesting the potential functional redundancy within the RDR gene family [35].

Within the AGO gene family, *KoAGO1*, *KoAGO4a*, and *KoAGO4b* showed an upregulation trend at several time points during low-temperature treatment, especially in the early stages of cold stress. This suggests that these genes might play key roles in the rapid response of *K. obovata* to cold stress through small RNA-mediated regulatory pathways [36]. Interestingly, *KoAGO10a* exhibited high expression at all recovery stages, even showing specific high expression, further suggesting that the AGO gene family may be involved in cold stress signal transduction and adaptation mechanisms in *K. obovata*. In response to low-temperature stress, we observed a fascinating phenomenon in the DCL gene family: all members displayed specific high expression during the early recovery stages following cold stress, making this an excellent entry point for further research. The RDR gene family exhibited a diversified expression pattern under cold stress. Similar to the DCL gene family, they showed significant expression during the early stages of cold stress, with specific expression observed in genes such as *KoRDR1b* during the later stages of cold stress.

Both the AGO and DCL families exhibited similar patterns in response to saline-alkali stress, with increasing expression of different members as the concentration of salt-alkali in the environment increased, a trend that was particularly pronounced in the DCL family. Many genes in the RDR gene family also showed high expression under different concentrations of salt-alkali stress, indicating that these genes may be widely involved in signal sensing and response to saline-alkali stress through small RNA-mediated regulatory networks [37]. Similar to the low-temperature stress response, all members of the DCL gene family displayed specific high expression during the mid-phase of waterlogging stress. Given their high expression in roots, it is clear that they play roles in maintaining root responses to hypoxia through small RNA-mediated regulatory pathways [38]. Similarly, *KoAGO5* and *KoAGO10b* exhibited enhanced root-specific high expression, suggesting their special roles in root adaptation to hypoxia.

### 3.5 Analysis of the protein functional regulatory network

**Fig. 7:**
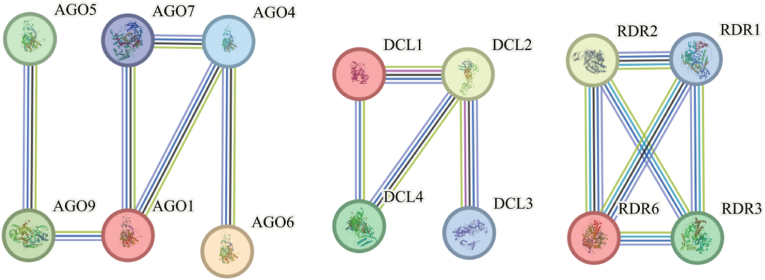
Protein functional regulatory network. AGO1, AGO5, AGO7, and AGO9 are all involved in RNA-mediated post-transcriptional gene silencing (PTGS). DCL1, DCL2 and DCL3, Ribonuclease (RNase) III involved in RNA-mediated post-transcriptional gene silencing (PTGS). DCL4, Ribonuclease (RNase) III involved in RNA-mediated post-transcriptional gene silencing (PTGS). RDR1, RNA-dependent direct polymerase involved in antiviral silencing. RDR2, involved in transcriptional gene silencing (TGS). RDR3, probably involved in the RNA silencing pathway and required for the generation of small interfering RNAs (siRNAs). RDR6, RNA-dependent RNA polymerase involved in post-transcriptional gene silencing (PTGS).

A comparative analysis of the protein interaction networks of the AGO, DCL and RDR gene families in *K*. *obovata* and *A*. *thaliana* can reveal the conservation and functional divergence of these genes in the RNA interference (RNAi) pathway (Fig. 7). The interactions of specific *K. obovata* members may reflect unique mechanisms of stress adaptation, such as low-temperature stress, by enhancing the connectivity of key proteins like AGO1, DCL2, and RDR6 to optimize small RNA synthesis or gene silencing functions. By integrating the protein interaction networks of the AGO, DCL and RDR gene families in *K. obovata* and *A*. *thaliana* with their gene expression data under low-temperature treatment, we unveiled the conservation and functional divergence of these genes in the RNAi pathway. Low-temperature stress significantly affected the expression levels of several key genes, and these changes were consistent with the regulatory properties of their interaction networks. This suggests that these genes may play a role in cold adaptation mechanisms by optimizing small RNA synthesis and gene silencing functions. These findings provide critical theoretical insights for further validation of the core roles of these genes in stress responses and for exploring their potential adaptive evolutionary processes.

### 3.6 Prediction and Analysis of the tertiary structure of K. obovata

**Fig. 8:**
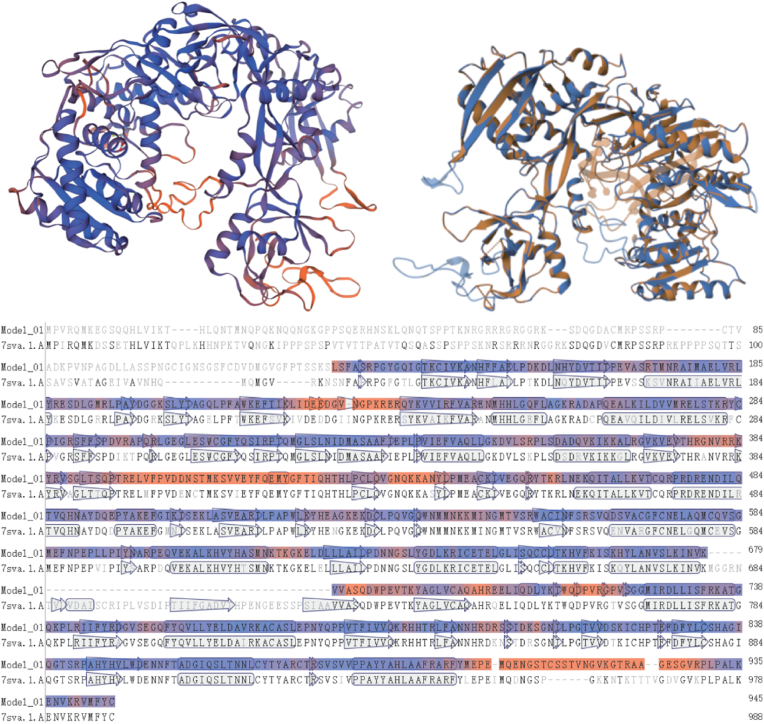
They are prediction structure diagram, comparison structure diagram and sequence comparison diagram respectively. Among them, the blue sequence part represents the confidence above 70 %, and the other colors represent the confidence between 60 and 70 %. The arrow shape represents β-sheets, and the cylinder shape represents α-helices.

We used the SWISS-MODEL online homology modeling platform to predict the tertiary structure of these proteins. Taking *KoAGO10a* with the best-fitting model as an example, we constructed the protein 3D structure and compared it with the template gene (7sva) (Fig. 8). Through structural comparison with closely related plants, we identified the secondary structures of *KoAGO10a*, such as α-helices and β-sheets. The high sequence similarity indicates that *KoAGO10a* shares a similar RNA regulatory function with 7sva. The figure shows the conserved regions between *KoAGO10a* and 7sva. These conserved regions are typically associated with RNA recognition, cleavage, or binding to siRNAs and miRNAs. Due to the highly conserved domains (PAZ and Piwi) present in *KoAGO10a*, it is likely involved in RNA-mediated gene silencing or post-transcriptional regulation processes [39]. In some non-conserved regions, the sequence differences between *KoAGO10a* and 7sva may affect its RNA binding ability, secondary structure formation, or interactions with other proteins [40]. In fact, *KoAGO10a* exhibits different secondary structures from 7sva, and these sequence variations can provide insights into the specific functions or possible evolutionary adaptations of *KoAGO10a*. By combining the expression patterns of *KoAGO4b*, we can more precisely reveal the potential role of *KoAGO10a* in specific biological processes.

## 4 Discussion

This study systematically identified the AGO, DCL and RDR gene families in the mangrove species *K. obovata* and *K. candel,* and analyzed *K. obovata* ’s expression patterns under different tissues and environmental conditions. Through gene sequence alignment and structural analysis, we found that they contain 8/6 AGO, 4/3 DCL and 5/4 RDR genes, with each gene family showing structural conservation and specific molecular functions. The two species are highly similar in evolution, but their differences may hold the key to *K. obovata* ‘s greater cold resistance.

### *4.1* *Argonaute proteins in K. obovata* and *K. candel*

Argonaute proteins can accommodate small RNA components and coordinate downstream gene silencing events through interactions with other protein factors [41,42]. In this study, we examined all three subfamilies of AGO genes in the genomes of *K. obovata* and *K. candel*. Based on a phylogenetic analysis of AGO genes from *A. thaliana*, *G. max*, and *V. vinifera*, we observed that monocots and dicots are clearly separated into different branches within the same subfamily. Notably, this conserved classification pattern within the *WRKY* gene family exhibits broad phylogenetic prevalence. Both monocots and dicots display minimal divergence in these groupings, retaining evolutionarily conserved functional activity [43,44]. This suggests that the divergence of AGO genes occurred prior to the split between monocots and dicots [45]. Interestingly, *KoAGO5*, *KcAGO5*, *KoAGO10a*, *KcAGO10a*, *KoAGO10b*, and *KcAGO10b* all cluster together within the same branch, implying that these homologous genes resulted from a recent duplication event. Gene duplication is a crucial factor in the evolution and expansion of gene families [[46], [47], [48]]. Previous studies have suggested that these genes may have played a similar role in the evolution of dicot plants. Our study revealed that, despite exhibiting highly similar evolutionary patterns, these sister species have significantly fewer AGO gene family members compared to other plants. This phenomenon may reflect either selective gene loss under evolutionary pressures or environment-specific functional optimization in *Kandelia*, potentially enhancing efficiency through pathway simplification [49]. Furthermore, within the AGO2 and AGO4 subfamilies, we observed significant evolutionary divergence between these two sister species. This divergence may indicate that certain genes in *K. obovata* play a crucial role in its enhanced cold tolerance compared to *K. candel*.

### 4.2 Dicer-like proteins in K. obovata and K. candel

Dicer-like proteins are key components in small RNA biosynthesis and play an important role in triggering transcriptional and post-transcriptional gene silencing in eukaryotes [5]. This study found that the DCL gene family in plants is highly conserved in terms of evolution, based on an evolutionary relationship analysis of *K. obovata* and *K. candel* along with model plants. The DCL gene family shows substantial similarity in both monocots and dicots, leading us to infer that they may share similar selection pressures and evolutionary models [50,51]. MEME analysis visually demonstrated that genes within the same subfamily share highly similar structural features. However, it is noteworthy that in DCL2, *KcDCL2* contains an additional Motif14 compared to *KoDCL2*. Despite this, the conservation of these genes remains evident, and this conservation is critical for the subfamily-specific functions [52]. Nevertheless, reports on the genetic analysis of the DCL gene family are limited, particularly with regard to the fact that *K. obovata* has a DCL3 member (*KoDCL3*) that *K. candel* lacks. This could be key to understanding why *K. obovata* demonstrates superior cold tolerance, warranting further investigation.

### 4.3 RNA-dependent RNA polymerase proteins in K. obovata and K. candel

RNA-dependent RNA polymerases are among the most widely used enzymes in RNA viruses, being indispensable for genome replication and transcription [53]. In this study, we identified the RDR gene family in these plants, and phylogenetic analysis indicated that, unlike *G. max* and *V. vinifera*, *K. obovata* and *K. candel* follow a relatively independent evolutionary pattern, with gene duplication events occurring between the two species. This may suggest that, throughout their long evolutionary history, *K. obovata*, an ancient mangrove species, has faced selective pressures that differ significantly from those experienced by other plants [8,54]. Similar to the DCL gene family, MEME analysis revealed that these two sister species share a common ancestor with other plants in the RDR gene family, and the high degree of conservation further underscores the critical role of these genes. Furthermore, *KoRDR5*, a gene unique to *K. obovata* within the RDR3 subfamily, may serve as a key regulator underlying its enhanced cold tolerance.

### 4.4 Conserved evolution and regulatory roles of AGO, DCL and RDR genes in K. obovata under different stress

This study systematically analyzed the expression patterns of the AGO, DCL and RDR gene families in *K*. *obovata* across different tissues and under low-temperature, saline-alkali, and waterlogging stress conditions. The results demonstrate that these gene families play critical roles in the growth, development, and stress adaptation of *K. obovata*. Notably, the expression of AGO genes exhibited significant differences across tissues, with higher expression levels in stems and roots, suggesting their potential roles in stress resistance and developmental regulation in plants. DCL genes play a key role in small RNA production, and the changes in their expression during different developmental stages highlight their functional diversity in plant growth and viral defense [55]. Furthermore, the expression of RDR genes was significantly upregulated under environmental stress, indicating their potential regulatory role in enhancing the plant's resistance to extreme environmental conditions. The evolutionary differences between *K. obovata* and *K. candel* may explain the key genes responsible for the greater cold tolerance of *K. obovata* compared to its sister species, providing a theoretical foundation for future targeted in-depth studies.

In the AGO gene family, *KoAGO1*, *KoAGO4a*, and *KoAGO4b* exhibited high expression levels across multiple tissues (e.g., stems, roots, and flowers) and were significantly upregulated under all stress conditions. This suggests that these genes, as core components, may be widely involved in the fundamental physiological activities and rapid stress responses of *K. obovata* through small RNA-mediated regulatory networks [56]. Some genes exhibited pronounced tissue-specific expression. Certain genes displayed strong responses to specific stress conditions [57]. Additionally, *KoRDR2*, *KoRDR5*, and *KoRDR1a* played critical roles during the adaptation and recovery phases of stress responses, demonstrating the functional specialization and coordination within the RDR gene family across different stress stages. The RDR gene family exhibited high expression in leaves, stems, and roots, with multiple members showing coordinated upregulation under stress conditions. This indicates potential functional redundancy within the family, which may enhance the environmental adaptability of *K. obovata* through complementary mechanisms. The tissue-specific expression patterns of AGO and DCL families under different stress conditions further suggest their highly coordinated functional differentiation and interaction within small RNA-mediated regulatory networks [58].

These gene families in *K. obovata* demonstrate significant tissue specificity, stress responsiveness, and functional redundancy, underscoring their indispensable roles in plant development and stress adaptation. Through coordinated expression regulation and involvement in small RNA pathways, these gene families collectively support the growth and survival of *K. obovata* under extreme environmental conditions. These findings enhance our understanding of *K. obovata*'s adaptive mechanisms and provide important insights into the role of small RNA regulatory networks in plant stress biology.

### 4.5 Expression and regulatory mechanisms of protein interaction networks and prediction analysis of tertiary structures in K. obovata

Through the protein interaction network analysis with model organisms, we identified several key proteins serving as pivotal hubs that play crucial roles in *K*. *obovata*'s response to complex stress environments [59]. These proteins interact within intricate regulatory networks, collaboratively modulating the functions of *K*. *obovata*. This provides a theoretical foundation for further in-depth studies on these critical proteins. The prediction of tertiary structures provides a theoretical basis for further in-depth research on the potential roles and specific functions of key genes in the biological processes related to *K. obovata*'s resistance to low-temperature stress.

This study provides a theoretical foundation for understanding the molecular mechanisms of K. obovata and opens new avenues for its application in ecological restoration and environmental protection. Through genetic engineering technologies [60], we can utilize AGO, DCL and RDR genes to enhance the stress resistance of *K. obovata*, thereby improving its adaptability in extreme environments. This has significant implications for strengthening *K. obovata*'s role in ecological protection and restoration. Furthermore, future research can further verify the specific functions of these genes in the cold resistance of *K*. *obovata* and explore the interactions between genes and environmental factors [61], particularly the regulatory mechanisms under low-temperature stress. Through this study, we can provide new strategies for enhancing the productivity and environmental adaptability of *K. obovata* and other mangrove plants, as well as others.

## 5 Conclusions

In summary, this study identified members of the AGO, DCL and RDR gene families in the genome of *K*. *obovata* and *K. candel*. Promoter, exon-intron structure, and phylogenetic analyses revealed the conserved evolution of these gene families in *K*. *obovata*. Prediction and Analysis of the Tertiary Structure with the template gene and the expression analysis under Environmental stress and protein interaction network analysis with model plants highlighted the complex regulatory mechanisms of these gene families in response to stress and identified potential core proteins. Collectively, these findings provide a foundation for further functional characterization of these genes and a better understanding of the regulatory mechanisms underlying *K*. *obovata*'s adaptation to cold stress.

## Statement

During the preparation of this work, the authors utilized generative AI technologies solely for language polishing. The final content was rigorously reviewed and edited by the authors, who take full responsibility for all academic claims.

## Declaration of competing interest

The authors state that there is no conflict of interest.

## Data Availability

Data will be made available on request.
